# Phase I trial compensation: How much do healthy volunteers actually earn from clinical trial enrollment?

**DOI:** 10.1177/17407745211011069

**Published:** 2021-05-02

**Authors:** Jill A Fisher, Lisa McManus, Julianne M Kalbaugh, Rebecca L Walker

**Affiliations:** 1 University of North Carolina at Chapel Hill, Chapel Hill, NC, USA; 2 Wake Technical Community College, Raleigh, NC, USA

**Keywords:** Phase I trials, healthy volunteers, study compensation, financial incentive, research payment, ethics

## Abstract

**Background/aims:**

Financial compensation for research participation is a major focus of ethical concern regarding human subject recruitment. Phase I trials are sometimes considered to be a lucrative source of income for healthy volunteers, encouraging some people to become “professional guinea pigs.” Yet, little is known about how much these clinical trials actually pay and how much healthy volunteers earn from them.

**Methods:**

As part of a mixed-methods, longitudinal study of healthy volunteers, we required participants to complete clinical trial diaries, or surveys that captured detailed information about screening and enrollment in Phase I trials. Over a 3-year period, participants provided information online or via telephone about each clinical trial for which they screened (e.g. the clinic name, the study’s therapeutic area, the length of the trial, the number of nights spent in the clinic, and the study compensation), and whether they qualified for trial inclusion. Clinical trial diaries generated data about whether participants continued to screen for and enroll in clinical trials and how much money they earned from their participation.

**Results:**

131 participants routinely completed clinical trial diaries or confirmed that they had not screened for any new clinical trials. Together, these participants screened for 1001 clinical trials at 73 research facilities during a 3-year period. Overall, the median clinical trial compensation was US$3070 (range = US$150–US$13,000). Participants seeking new healthy volunteer trials tended to screen for three studies per year, participate in one or two studies, and earn roughly US$4000 annually. Participants who were unemployed earned the most income from clinical trials compared to those with full-time or part-time jobs, and those individuals whom we label “occupational” participants because of their persistent pursuit of clinical trials earned more than people who screened occasionally. Notably, the median annual trial compensation was well below US$10,000 for all employment groups, and most occupational healthy volunteers also earned less than US$10,000 each year. The 10% of participants who earned the most had a median annual income of US$18,885 from clinical trials, and there was significant volatility in these individuals’ earnings from year to year.

**Conclusion:**

Despite the perception that Phase I enrollment can generate significant earnings, it was exceedingly rare for anyone in this study to make more than US$20,000 in a single year, and unusual to earn even between US$10,000 and US$20,000. From an ethics perspective, individual trials might appear to unduly induce enrollment by offering significant sums of money, but given our findings, the larger problem for low-income participants may be the unrealistic perception that clinical trials alone could be a way of earning a living.

## Background/aims

Payment for research participation has long been a controversial topic.^1–5^ Phase I healthy volunteer trials—in which individuals are exposed to the risks of consuming investigational drugs with no possibility for medical benefit—typically offer more substantial payments than other types of research. Phase I trials also frequently require healthy volunteers to accept intense medical and behavioral monitoring, including confinement in a residential research facility, for days or weeks as part of their participation.^6^ This unique set of circumstances has spurred ethical concerns about unduly inducing participation with offers of high compensation, on one hand, and exploiting participants through offering low payments, on the other hand.^7–11^ The literature suggests that healthy volunteers can earn anywhere from a few hundred dollars to over US$10,000 from Phase I trials, depending primarily on the length of the study.^12,13^ Some healthy individuals even use Phase I trials as their sole source of income, creating a cadre of “professional guinea pigs” or “professional lab rats.”^14–16^ While apparently primarily a US phenomenon, other countries also report serial participation among healthy volunteers motivated to earn income through clinical trials.^17–19^

Despite significant attention to the ethics of Phase I trial compensation and concerns about so-called professional participation, little is known about how much these trials actually pay and how much healthy volunteers can expect to earn, either through occasional enrollment or more concerted effort to earn a living. In the United States, there are no centralized participant registries or limitations on how much income individuals can earn from medical research.^20^ By taking the scholarly and popular literature at face value, Phase I trials are a lucrative source of income, such as when it is suggested that healthy volunteers can earn over US$30,000 annually from clinical trials.^21–23^ A research participation website even suggests that healthy volunteers can “theoretically” enroll in six to seven trials and earn an average of US$18,000 to US$28,000 annually by “do[ing] studies for a living.”^24^ Examining Phase I participation is therefore crucial to providing insights into what can be expected as typical trial earnings, as well as the upper limits of what healthy volunteers might make when they pursue clinical trials persistently.

This article uses data from a longitudinal study of US healthy volunteers to document the clinical trial income that participants earned annually over a 3-year period. Because people have differing levels of clinical trial involvement, we provide details about the clinical trial activities (e.g. number of screenings, number of trials, and time spent in clinics) based on participants’ employment status (i.e. full-time, part-time, or unemployed) and their pursuit of clinical trials (i.e. occasional or occupational). We also compare the top-earning healthy volunteers to the others in this study. This empirically grounded snapshot of clinical trial compensation can subsequently shed light on which ethical concerns are the most salient in the context of Phase I trials.

## Methods

From 2013 to 2017, we conducted a mixed-methods, longitudinal study of individuals who were enrolled in Phase I trials as healthy volunteers. The purpose of this National Institutes of Health (NIH)-funded study was to investigate how healthy people perceive the risks and benefits of their clinical trial participation over time, how they make decisions about their trial enrollment, and how trial participation affects their health behaviors. A key study component was to collect data in real time about participants’ clinical trial involvement, including screenings and completed trials. The study was reviewed and approved by the Biomedical Institutional Review Board at the University of North Carolina at Chapel Hill (#13-1256).

Seven US Phase I clinics (three in the East, two in the Midwest, and two in the West) gave us permission to visit their facilities and recruit clinical trial participants. Other than providing access to participants, the clinics were not involved in study design, conduct, or analysis. Participants were recruited by the principal investigator (PI) and other study team members between May and December 2013. All individuals who were enrolled as healthy volunteers at one of these research clinics and spoke English or Spanish were eligible for this study. To incentivize enrollment, participants were given a US$20 Visa gift card after enrollment and completion of an initial interview and could earn up to US$450 more over 3 years by completing this study. Approximately, 90% of individuals invited to participate subsequently enrolled. All participants provided written informed consent.

The study design included “full-participation” and “control” arms to which participants were randomized in a 4:1 ratio after enrollment. The full-participation arm was interviewed a total of five times (in person at enrollment, then via telephone 6 months, 1 year, 2 years, and 3 years later) and completed clinical trial diaries (CTDs), which were surveys that captured detailed information about clinical trial screening and enrollment. Control participants were interviewed twice (in person at enrollment, then via telephone 3 years later) and did not complete CTDs. The purpose of the control group was to assess whether this study unintentionally affected how often participants enrolled or how they perceived Phase I trials. At enrollment, we collected participant demographic information, including gender, race, ethnicity, and age. Additional demographics such as employment status, current job, educational attainment, and household income were updated at each interview stage.

Participants accessed the CTD instrument online by logging into a survey portal. A shorter version was also available for participants to complete via telephone with a study team member. The CTD collected information about each clinical trial for which the person screened (e.g. the clinic name, screening date, trial start date, the trial’s therapeutic area, study compensation, trial length, and the number of nights spent in the clinic) and whether they qualified for trial inclusion. If they qualified and enrolled, the CTD collected data about their experiences in that trial.^13^ Participants were required to complete a CTD every time they screened, but they were not obligated to screen for any clinical trials while enrolled in this study and were not compensated for completing CTDs. We contacted participants by email, text, and/or phone every 1–3 months reminding them to either complete CTDs or let us know they had not screened for any trials. We also used scheduled interviews to check for missing CTDs and then updated their CTD record.

## Results

### Sample population

We enrolled 180 healthy volunteers in this study. This sample size ensured adequate representation of participant diversity while maintaining feasibility for the study’s intensive qualitative portion. A total of 146 healthy volunteers were randomized to the study’s full-participation arm, and we retained 133 (91.1%) through the entire 3-year protocol. Of those not retained, nine were lost to follow-up (including one death), three voluntarily withdrew, and one was withdrawn by the study team. For the study’s quantitative portion, 131 participants routinely completed CTDs or confirmed that they had not screened for any new clinical trials, so the current analysis is based on those 131 individuals’ CTD data. (Thirty-four participants were allocated to the control arm, and 33 (97.1%) were retained. They are not included here because this arm did not complete CTDs.)

Our sample was recruited from the seven Phase I clinics to achieve equal representation across the Eastern, Midwestern, and Western United States. Table 1 presents participants’ demographic characteristics, and the sub-sample of 131 participants largely reflects both our overall sample (full-participation and control arms) as well as other empirical studies of healthy volunteers.^25–27^ Three-quarters of participants in our sample were men, and two-thirds were from racial and ethnic minority groups. Almost half were at least 40 years old at enrollment, and 81% were 30 or older. Nearly 70% of participants did not have a college degree, and over 40% reported an annual household income (including clinical trial compensation) of less than US$25,000. At the time of enrolling in this study, 55% of participants had completed five or more clinical trials, and only 20% were in their first trial.

**Table 1.** Study participants’ demographics characteristics and clinical trial activity (N = 131).

	n	%
Gender		
Women	32	24.4%
Men	99	75.6%
Race/ethnicity		
Non-Hispanic Black/African American	49	37.4%
Non-Hispanic other^a^	8	6.1%
Non-Hispanic White	45	34.4%
Hispanic^b^	29	22.1%
Age at enrollment		
18–21	3	2.3%
22–29	22	16.8%
30–39	47	35.9%
40–49	39	29.8%
50+	20	15.3%
Educational attainment at enrollment		
Less than high school	9	6.9%
High school or GED	28	21.4%
Some college	40	30.5%
Trade/technical/vocational training	14	10.7%
Associate’s degree (2-year college degree)	12	9.2%
Bachelor’s degree (4-year college degree)	25	19.1%
Graduate degree	3	2.3%
Household income at enrollment^c^		
Less than US$10,000	20	15.3%
US$10,000–US$24,999	36	27.5%
US$25,000–US$49,999	55	42.0%
US$50,000–US$74,999	11	8.4%
US$75,000–US$99,999	4	3.1%
US$100,000 or more	4	3.1%
Clinical trial experience at enrollment		
1 study	27	20.6%
2–4 studies	32	24.4%
5–10 studies	37	28.2%
11–200 studies	35	26.7%
Clinical trial screenings after enrollment		
No new screenings	16	12.2%
1–5 new screenings	49	37.4%
6–10 new screenings	33	25.2%
11 or more new screenings	33	25.2%
Clinical trial participation after enrollment		
No new studies	31	23.7%
1–2 new studies	34	26.0%
3–5 new studies	33	25.2%
6–10 new studies	24	18.3%
11 or more new studies	9	6.9%

GED: General Educational Development.

a The category Non-Hispanic other includes individuals who identified as American Indian, Asian, Native Hawaiian/Pacific Islander, and more than one race.

b The category Hispanic includes all racial groups, of which we have those in our sample who identified as White, Black, more than one race, American Indian, and Native Hawaiian/Pacific Islander.

c Data for household income were not reported by one participant.

Our final data set included 1138 CTDs, each representing a participant’s attempt to enroll in a clinical trial. Excluding CTDs done for “baseline” clinical trials at enrollment in this study, 131 participants screened for 1001 subsequent trials over the next 3 years. These trials were conducted at 73 different US clinics, including academic research centers, pharmaceutical company clinics, contract research organization clinics, and independent research clinics (for more details about the clinical trials, see Supplemental Materials). Sixteen (12.2%) participants did not screen for any subsequent trials, but the vast majority screened for at least one more (median = 6; maximum = 53) (Table 1). Out of the 1001 new screenings, the participants did not qualify for those trials in 259 instances (25.9%). Participants enrolled in 497 (67.0%) of the remaining 742 clinical trials. In the other instances, participants decided not to enroll, were not selected to participate despite qualifying, were medically disqualified on the trial start day, or were notified the trial had been canceled. During the 3 years that we followed participants, 31 (23.7%) did not complete another trial, including both the participants who never screened again and 15 who screened but did not enroll in any new trials. Of the remaining participants, roughly a quarter enrolled in one or two new trials, a quarter enrolled in three to five new trials, and the final quarter enrolled in six or more new trials (median = 3; maximum = 22) (Table 1).

### Financial compensation offered

Participants’ CTD data provide a snapshot of how much financial compensation US Phase I trials offer healthy volunteers. Our participants screened for clinical trials with a wide range of study payments. The smallest was a US$150 vaccine study that did not require any clinic confinement; whereas the largest was a US$13,000 cancer study that required healthy volunteers to spend 34 consecutive days and nights in the research clinic. Overall, the median compensation was US$3070, and the majority of trials (65.1%) offered less than US$4000, with fewer than 2% offering compensation above US$10,000 (Table 2).

**Table 2.** Distribution of available clinical trials by pay.

Study pay categories	Total number of studies (%)	Median pay per day enrolled (IQR)
US$0–US$1999	221 (22.9%)	US$135 (184)
US$2000–US$3999	409 (42.3%)	US$168 (148)
US$4000–US$5999	194 (20.1%)	US$215 (118)
US$6000–US$7999	87 (9.0%)	US$233 (95)
US$8000–US$9999	39 (4.0%)	US$246 (75)
US$10,000–US$11,999	10 (1.0%)	US$293 (114)
US$12,000–US$13,999	7 (0.7%)	US$263 (100)
Grand total*	967	US$196 (153)

IQR: interquartile range.

* Post-baseline studies that were missing data about study payment and studies with a length of more than 120 days are not included.

The median clinical trial length, regardless of payment, was 20 days, with a median clinic confinement of nine nights. At times, these were consecutive nights, but, in other instances, participants would come and go for multiple confinement periods. (We refer to the confinement period as “nights” to indicate that participants were required to sleep and normally spend a 24-h period in the clinic.) In general, the longer the trial, the more compensation offered to participants. We also calculated the trials’ daily rate by dividing the total compensation by the trial length, and the median rate was US$196 per day (Table 2). Importantly, the calculated daily rate is a crude metric of time in the trial, without accounting for whether participants needed to be at the clinic (n.b. they would be expected to follow the trial protocol regardless of whether they were in the clinic). Although there was fluctuation across the study payment bands, the higher the compensation amount, the higher the daily median pay, except for the studies paying more than US$12,000. Based on the interquartile ranges, the most variability in the daily compensation rate was in the lowest-paying studies, particularly those offering less than US$2000. In sum, some clinical trials available to healthy volunteers offered rather substantial payment, but the plurality of clinical trials compensated between US$2000 and US$4000.

### Annual clinical trial activity and earnings

Following a cohort of healthy volunteers for 3 years allowed unprecedented insight into serial clinical trial participation, as well as attrition from participation. At enrollment in this study, every participant was necessarily a current healthy volunteer in a clinical trial. One year later, 86.3% of participants continued to screen; the next year, 67.2% continued to screen; just 52.3% screened in the third year (Table 3). Despite the decline in trial participation overall, clinical trial activity among those who were seeking new trials was quite consistent across the 3 years. In general, these participants tended to screen for three trials, participate in one to two trials, and earn roughly US$4000 annually (Table 3). In 75.1% of the cases (counted as each participant’s annual income for each of 3 years), participants who screened for at least one trial earned less than US$10,000 in study compensation in a given year; indeed, 19.7% earned no income, 34.6% earned less than US$5000, and only 20.8% earned between US$5000 and US$9999. Participants earned annual compensation of between US$10,000 and US$19,999 in 17.5% of cases, and over US$20,000 in just 7.4% of cases.

**Table 3.** Healthy volunteers’ earnings and participation over 3 years.

	Number ofparticipants		Screeningsper personper year		Participationper personper year		Pay per personper year (US$)	
	*Total*	*n whoscreened (%)*	*Median(minimum–maximum)*	*IQR*	*Median (minimum–maximum)*	*IQR*	*Median(minimum–maximum)*	*IQR*
*Baseline to year 1*	131	113 (86.3%)	3 (1–16)	3	2 (0–9)	2	3950 (0–47,200)	6720
*Year 1 to year 2*	131	88 (67.2%)	3 (1–22)	3	1 (0–8)	2	4100 (0–32,800)	9059
*Year 2 to year 3*	130	68 (52.3%)	3 (1–15)	5	1 (0–8)	2	4800 (0–39,605)	9824

IQR: interquartile range.

Healthy volunteers’ overall financial situations vary, so we queried how our participants’ employment status mapped onto their clinical trial activity and earnings (Table 4). Employment fluctuated by year, with people moving among full-time, part-time, and no formal work. Attrition from clinical trial participation was most marked in the full-time and unemployed categories, while the percentage of individuals with part-time employment seeking clinical trials remained relatively more stable. Of those actively pursuing trials, individuals with full-time jobs typically screened and participated less often than did individuals who worked part-time, who in turn screened and participated less often than did individuals who were unemployed. The average clinical trial length—as a function both of total days enrolled and nights confined to a research clinic—was similar across employment groups. However, with more trial enrollment, unemployed participants generally spent a greater total amount of time annually in clinical trials than the other two groups. Regarding study payment, full-time workers typically earned US$2350–US$4400 from enrolling in one trial per year, part-time workers typically earned US$3850–US$5400 from enrolling in one to two trials per year, and unemployed people typically earned US$5638–US$12,525 from enrolling in two to four trials per year. Thus, healthy volunteers who were unemployed earned the most income from clinical trials compared to those with full-time or part-time jobs. More broadly, it is striking that except for unemployed individuals who participated in clinical trials during the final study year, the median annual trial compensation was well below US$10,000 for all employment groups.

**Table 4.** Healthy volunteers’ earnings and participation over 3 years by employment status.

	Number ofparticipants		Screenings perperson per year		Participation perperson per year		Total time inclinical trials^a^				Pay per person peryear (US$)	
	*Total*	*n whoscreened (%)*	*Median(minimum–maximum)*	*IQR*	*Median (minimum–maximum)*	*IQR*	*Median days(minimum–maximum)*	*IQR*	*Median nights(minimum–maximum)*	*IQR*	*Median(minimum–maximum)*	*IQR*
	*Baseline to year 1*											
*Full-time*	55	43 (78.2%)	2 (1–8)	3	1 (0–6)	2	44 (4–188)	55	13 (0–116)	22	2350 (0–31,425)	5400
*Part-time*	34	30 (88.2%)	3 (2–12)	3	2 (0–9)	2	45 (3–216)	64	17 (1–142)	12	5400 (0–47,200)	6498
*Unemployed*	42	40 (95.2%)	4 (1–16)	3	2 (0–8)	3	46 (3–365)	91	24 (2–124)	61	5638 (0–33,607)	11,428
	*Year 1 to year 2* ^b^											
*Full-time*	60	33 (55.0%)	2 (1–7)	2	1 (0–4)	1	31 (3–99)	47	12 (0–40)	22	2500 (0–15,000)	6825
*Part-time*	35	27 (77.1%)	4 (1–10)	4	1 (0–5)	1	50 (8–158)	87	11 (0–65)	25	3970 (0–18,815)	6925
*Unemployed*	35	28 (80.0%)	4 (1–22)	4	2 (0–8)	3	65 (10–175)	86	35 (2–120)	53	6500 (0–32,800)	16,062
	*Year 2 to year 3*											
*Full-time*	64	23 (35.9%)	1 (1–10)	2	1 (0–7)	1	37 (5–365)	47	19 (3–65)	24	4400 (0–21,455)	6200
*Part-time*	35	25 (71.4%)	3 (1–15)	5	1 (0–6)	3	80 (14–365)	82	17 (1–71)	27	3850 (0–19,450)	9150
*Unemployed*	31	20 (64.5%)	5 (1–15)	6	3.5 (1–8)	4	97 (2–251)	110	42 (2–138)	68	12,525 (500–39,605)	17,504

IQR: interquartile range.

a We included only data here for the clinical trials that participants actually did. For those who screened but did not participate, they did not spend any days enrolled or night in the clinic, and eliminating those cases from the aggregate for this category allows better comparison across employment groups.

b We are missing data about one participant’s employment status at this timepoint, so have not included him in the analysis.

Phase I trial participation can be categorized as either “occasional” or “occupational” depending on the healthy volunteer’s orientation to trial enrollment. Occupational participants are a designation we gave to those who reported focusing on clinical trials as their primary source of income and pursuing studies as though it were a full-time job. Most occupational participants were otherwise unemployed but some held part-time jobs. Occasional participants were less focused on enrolling in trials than occupational participants, and they included individuals who were unemployed or held part-time jobs, as well as all participants who held full-time jobs. Most people were occasional participants, and the occupational category decreased from a peak of 45 participants (34.3%) at enrollment in this study down to 31 (23.7%) 1 year later, 29 (22.1%) 2 years after enrollment, and only 18 (13.8%) 3 years after enrollment at this study’s end (Table 5).

**Table 5.** Healthy volunteers’ earnings and participation over 3 years by occasional versus occupational participation.

	Number ofparticipants		Screenings perperson per year		Participation perperson per year		Total time inclinical trials^a^				Pay per personper year (US$)	
Participanttype	*Total*	*n whoscreened (%)*	*Median(minimum–maximum)*	*IQR*	*Median (minimum–maximum)*	*IQR*	*Median days(minimum–maximum)*	*IQR*	*Median nights(minimum–maximum)*	*IQR*	*Median(minimum–maximum)*	*IQR*
	*Baseline to year 1*											
*Occasional*	100	82 (82%)	2 (1–8)	1	1 (0–6)	2	32 (3–188)	37	13 (0–116)	15	2650 (0–31,425)	5381
*Occupational*	31	31 (100%)	5 (1–16)	4	4 (1–9)	2	88 (4–365)	77	32 (3–142)	48	9700 (2720–47,200)	11,945
	*Year 1 to year 2*											
*Occasional*	102	59 (57.8%)	2 (1–7)	2	1 (0–4)	1	31 (3–158)	48	8 (0–40)	22	2500 (0–15,000)	5600
*Occupational*	29	29 (100%)	5 (2–22)	3	3 (0–8)	3	83 (8–175)	80	33 (3–120)	50	9017 (0–32,800)	13,646
	*Year 2 to year 3*											
*Occasional*	112	50 (44.6%)	2 (1–10)	2	1 (0–7)	2	42 (2–365)	68	16 (1–65)	24	3340 (0–21,455)	8235
*Occupational*	18	18 (100%)	7 (3–15)	5	4.5 (1–8)	2	111 (34–251)	102	53 (2–138)	59	16,425 (2235–39,605)	12,073

IQR: interquartile range.

a As with Table 4, we included only data here for the clinical trials that participants actually did.

In terms of clinical trials, occupational participants screened each year whereas the occasional participants did not always do so. Occupational participants also spent more time annually in trials compared to occasional participants because they participated in more trials per year, but the per-trial time commitment was quite similar between the groups. As expected, occupational participants earned considerably more money from clinical trials each year (US$9017–US$16,425 compared to US$2500–US$3340). However, in only 1 year of this study did the annual median trial income for occupational participants top US$10,000. Indeed, the range of annual incomes that participants generated indicates that some occasional participants even earned *more* than some occupational participants; the primary difference was that the range included higher amounts of compensation among the occupational participants (maximum total income over each of the 3 years of US$15,000–US$31,425 for occasional participants compared to US$32,800–US$47,200 for occupational participants).

Although they are not representative of all healthy volunteers, we summarize in Table 6 the clinical trial activity of the top 10% of earners, most of whom were categorized as occupational participants during our entire study. Notably, there was a striking difference in 3-year total compensation between the top earner (i.e. US$100,700) and the participant at the bottom of this small group’s rankings (i.e. US$33,550). In aggregate, these 14 participants screened for 317 clinical trials and participated in 192, equaling a median of 6 screenings, 4 trials, and income of US$18,885 annually. Comparing participants’ study compensation across each study year, the high volatility in income from trials becomes apparent, even among the highest earners. Specifically, the top earner’s income ranged from US$28,295 to US$39,605, and the second highest earner had a low of US$17,050 and a high of US$47,200.

**Table 6.** Clinical trial activity over 3 years of the top 10% earners.

	All years	Year 1					Year 2					Year 3				
Ranking	*Total earnings*	*Newscreenings/studies*	*Newearnings*	*Days enrolled/nights in clinic*	*Average earningsper trial day*	*Distancetraveled(miles)*	*Newscreenings/studies*	*Newearnings*	*Days enrolled/nightsin clinic*	*Averageearningsper trial day*	*Distancetraveled(miles)*	*Newscreenings/studies*	*Newearnings*	*Days enrolled/nights in clinic*	*Averageearningsper trial day*	*Distancetraveled(miles)*
1	100,700	16/5	28,295	131/108	216	11,168	22/8	32,800	138/120	238	18,063	15/7	39,605	215/138	184	12,952
2	92,150	10/9	47,200	216/142	219	7973	7/5	17,050	106/65	161	1186	10/8	27,900	182/86	153	2772
3	89,930	6/5	24,550	118/98	208	5093	5/4	30,500	122/91	250	15,769	5/5	34,880	138/106	253	10,408
4	75,502	15/8	33,607	191//111	176	11,561	13/6	22,445	103/82	218	10,511	15/5	19,450	90/71	216	9293
5	59,270	4/4	13,570	62/60	219	1494	5/4	22,600	86/82	263	1837	5/5	23,100	81/52	285	2518
6	58,915	5/4	13,630	79/49	173	5272	11/7	26,385	175/69	151	8526	11/7	18,900	117/53	162	4929
7	56,500	5/4	28,500	111/95	257	12,345	4/3	7500	39/35	192	594	5/4	20,500	94/92	218	1682
8	55,425	9/5	18,870	164/69	115	6854	7/3	15,100	86/57	176	8206	10/7	21,455	365/65	59	9460
9	54,761	4/4	31,419	132/124	238	7002	4/3	17,842	65/61	274	4666	1/1	5500	21/20	262	2503
10	50,575	11/5	13,475	88/42	153	1878	6/4	18,600	114/59	163	1162	14/6	18,500	127/63	146	25,551
11	42,325	6/6	31,425	124/116	253	5291	3/2	5200	28/26	186	1197	3/2	5700	31/24	184	1632
12	41,425	4/3	15,725	72/70	218	1106	4/3	10,700	46/25	233	582	3/2	15,000	43/41	349	1093
13	40,425	5/4	19,100	116/62	165	10,460	6/3	12,225	91/32	134	1040	6/3	9100	75/16	121	1007
14	33,550	6/2	5900	46/23	128	5591	3/3	10,450	61/31	171	15,448	8/4	17,200	109/60	158	16,041
**Median top 10%**	**55,963**	**6**/**4.5**	**21,825**	**117**/**82.5**	**187**	**6222**	**5.5**/**3.5**	**17,446**	**88.5/60**	**197**	**3251**	**7**/**5**	**19,175**	**101.5**/**61.5**	**189**	**3850**
**Median other 90%**	**5500** ^a^	**3**/**2**	**4713**	**38/13**	**152**	**319**	**3**/**1**	**4400**	**31/10**	**126**	**185**	**2**/**1**	**4800**	**60/16**	**110**	**190**

a Unlike the top 10% earners, not all of the other 90% screened for a clinical trial throughout the 3 years of the study. As a result, the median total earnings include individuals who did not pursue clinical trials in each of the 3 years.

To contextualize these higher earnings, Table 6 summarizes participants’ total annual days enrolled in trials, nights spent in research clinics, and the estimated distance traveled to screen and participate. The top earners spent a median of 104.5 days and 64 nights annually in clinical trials, with a high of 365 days for a participant who joined a lengthy Ebola vaccine trial and 142 nights for the participant who completed nine trials in 1 year. The top earners joined trials with a wide range of daily rates (low = US$59/day; high = US$349/day), indicating that they did not enroll only in studies that paid the most for their time. In addition, the top earners traveled a median distance of 5196.5 miles annually for clinical trials, with one participant logging 25,563 miles in a single year when he screened for studies throughout the Southwestern and Midwestern US. While it was not just the top earners who spent a large portion of the year participating in and traveling for trials, these individuals generally spent more time in trials and traveled further to enroll. As point of comparison, the other 90% of this study’s participants spent a median of 38 days and 13 nights in trials annually while traveling a median distance of just 217 miles.

## Limitations

Our findings are based on participants’ self-reported clinical trial activity over a 3-year period, which is subject to inaccuracies or missing data. To improve data quality, we contacted participants frequently to ask whether they had screened for any new trials, and we asked probing questions during our regularly scheduled interviews to check our records and collect data for any missing CTDs. Participants generally exhibited excellent recall about trial compensation and nights in the clinic, whereas other details, particularly about the drugs’ therapeutic target (see Supplemental Materials), were more prone to missing or uncertain information, especially when they screened but did not participate in a trial. Participants also erred on overreporting, such as by completing CTDs for focus groups, marketing studies, or non-interventional medical studies they had done. We excluded those studies from our data set, but they confirm most participants’ thoroughness in providing information to the study team.

Another potential limitation of this study stems from the concern that involvement in the full-participation arm could have affected participants’ clinical trial activity. The control arm participants did not complete CTDs, so we simply asked during their final interview how many clinical trials they had screened for and participated in during the previous 3 years. This data collection method is likely less reliable than CTDs, particularly for people who screened frequently. However, including all retained participants (regardless of whether they screened each year), the median number of screenings for the entire 3-year period was 6 for both the control and full-participation arms (IQR = 9 for both), and the median number of trials was 3.5 for the control arm and 3 for the full-participation arm (IQR = 7 for control and 5 for full-participation arm). Therefore, completing CTDs and being in this study’s full-participation arm did not appear to affect participants’ clinical trial activity.

## Conclusion

Assumptions about Phase I trial earnings drive ethical concerns both about paying healthy volunteers too much and too little. Yet, data about such income are sparse. This empirical study focused on the question of how much healthy volunteers actually earn from their trial participation. We found a large payment spectrum for Phase I trials, ranging from US$150 to US$13,000 per trial. Considerably more trials paid less than US$2000 (22.9%) or between US$2000 and US$4000 (42.3%) as compared to trials paying more than US$6000 (14.7%). Participants earned a median of US$4200 annually from trials, with wide variability from year to year as well as among participants.

Employment influenced trial participation such that, in aggregate, unemployed individuals had the highest rates of participation and trial earnings, followed by people with part-time jobs, then those with full-time employment. Occupational healthy volunteers, whom the literature might consider “professionals,” also participated and earned more than occasional participants. Nonetheless, we found it to be exceedingly rare for anyone to make more than US$20,000 in a single year, and even unusual to earn US$10,000–US$20,000. For participants who rely solely on clinical trials for their annual income, these levels of compensation typically generate below poverty-line income. Thus, while trial compensation may supplement one’s income or help make ends meet, it is not a mechanism to achieve financial security.^28–30^

This study’s longitudinal design also helps characterize serial enrollment in clinical trials, both in terms of persistence in and attrition from trials over 3 years. Just 52.3% of our sample screened for any Phase I trials in the study’s final year. While this was true across employment categories, part-time workers had the most stable levels of trial participation relative to full-time workers and unemployed people. Their continued trial involvement could be because they had enough income to more reliably afford the transportation expenses related to screening (compared to the unemployed), but not enough money to otherwise make ends meet (compared to full-time workers). Trial participation is not guaranteed, and study participants completed only two-thirds of the trials for which screened. Not only are there costs associated with screening, such as travel costs or missed wages from a job, but individuals also might become discouraged from future participation when they have difficulty qualifying for studies. Even the number of occupational participants declined over time, with some becoming occasional participants and others no longer screening at all. The occupational participants in the final study year made a considerably higher median income than in the prior 2 years (US$16,425 compared to US$9017–US$9700), which might indicate that those who continued this mode of trial participation were the most financially successful at it. In sum, our results suggest that paid clinical trial participation—whether as occasional or even “professional” healthy volunteers—appears to be a relatively short “career.” The CTD data cannot provide an explanation for *why* healthy individuals might discontinue their trial participation, but this is an area ripe for future qualitative research.

Data from our sample’s top 10% of earners reveal the upper limits of what income might be possible from clinical trials. One participant earned US$47,200 in a single year, but there was high volatility in his 3-year earnings. Overall, these data indicate that even occupational participation results in quite low annual earnings. The top 10% earners’ median annual income was US$18,885, and some of these participants earned less than US$10,000 in 1 or even 2 of the 3 years we followed them. On average, the top earners participated in trials that paid higher daily rates than the other participants. However, because median daily rates varied dramatically, the key component of the top earners’ ability to generate larger amounts of trial compensation was the considerable effort they invested in trials. Compared to the other 90% of participants, the top earners were enrolled in trials for a sizable portion of the year, spent much of the year living and sleeping in a research facility, and logged hundreds or thousands of miles to screen for and enroll in trials. Thus, the top earners were not outperforming the other participants by simply picking the highest-paying clinical trials.

The study findings suggest that clinical trial participation is not as lucrative for healthy volunteers as the literature often implies. The so-called professionals also make up the minority of trial participants, despite the scholarly attention paid to this group.^14–16^ Exceedingly few participants were able to earn over US$30,000 annually, which has been suggested as a potential income level.^21,23^ Even the more modest estimate of US$18,000–US$28,000 in annual earnings given on a website for aspiring “professional lab rats” may set unreasonable expectations for all but the most committed healthy volunteers.^24^ Indeed, among top earners, no one made more than US$30,000 annually in all 3 years of this study, and it was not uncommon for even these individuals to earn below US$18,000 in a single year. Significantly, many clinics do not offer financial compensation for screening, and few reimburse participants’ transportation and other expenses, so participants incur financial risk in screening for new clinical trials, particularly when they travel to do so.

Examining Phase I trial participation in an empirically grounded way can inform ethical attention to payments for healthy volunteer studies. Worries about unduly inducing trial participation through offers of substantial payment are contextualized by accounting for healthy volunteers’ employment status and overall trial income. While particular trials may offer sums of money that motivate enrollment in ethically dubious ways, the larger problem may be the perception that serial participation could be a lucrative job. Indeed, even the ethics literature has fallen prey to this assumption, leading to the hotly debated question about whether clinical trials should be considered a form of work.^31–36^ Given the relatively few participants who pursue Phase I trials occupationally, perhaps too much attention is paid to this small subset of healthy volunteers. Instead, ethical focus should be shifted to the question of what the paid research involvement of individuals with low-income and low-educational attainment might mean if they believe they can earn much higher annual incomes from serial participation than are truly possible. This question shifts the ethical frame away from undue inducement to participate in particular trials and toward concerns about exploitation by a system that appears to promise unrealistic financial rewards for healthy volunteers’ continued involvement. Our findings demonstrate that underemployed people of color are expending extensive effort to earn income through their clinical trial participation, and that even among the most dedicated participants, their earnings represent their economic precarity rather than providing a living wage.^37,38^

## Supplemental Material

sj-pdf-1-ctj-10.1177_17407745211011069 – Supplemental material for Phase I trial compensation: How much do healthy volunteers actually earn from clinical trial enrollment? Click here for additional data file. Supplemental material, sj-pdf-1-ctj-10.1177_17407745211011069 for Phase I trial compensation: How much do healthy volunteers actually earn from clinical trial enrollment? by Jill A Fisher, Lisa McManus, Julianne M Kalbaugh and Rebecca L Walker in Clinical Trials
