# Supplementary material for: Phase I trial compensation: How much do healthy volunteers actually earn from clinical trial enrollment?
Source: Clin Trials. 2021 May 2;18(4):477–87. doi: 10.1177/17407745211011069 (PMC8290991; doi:10.1177/17407745211011069)
Supplement: sj-pdf-1-ctj-10.1177_17407745211011069 – Supplemental material for Phase I trial compensation: How much do healthy volunteers actually earn from clinical trial enrollment? [file sj-pdf-1-ctj-10.1177_17407745211011069.pdf]

Supplemental Table 1. Number of Research Clinics by Region (N = 73)

Clinic Region	n	%
Florida	4	5.5%
Lower Great Lakes	9	12.3%
Midwest	6	8.2%
Mountains	3	4.1%
North Central	2	2.7%
Northeast	27	37.0%
Northwest	0	0.0%
Southeast	3	4.1%
Southwest	10	13.7%
Texas	5	6.8%
Upper Great Lakes	4	5.5%

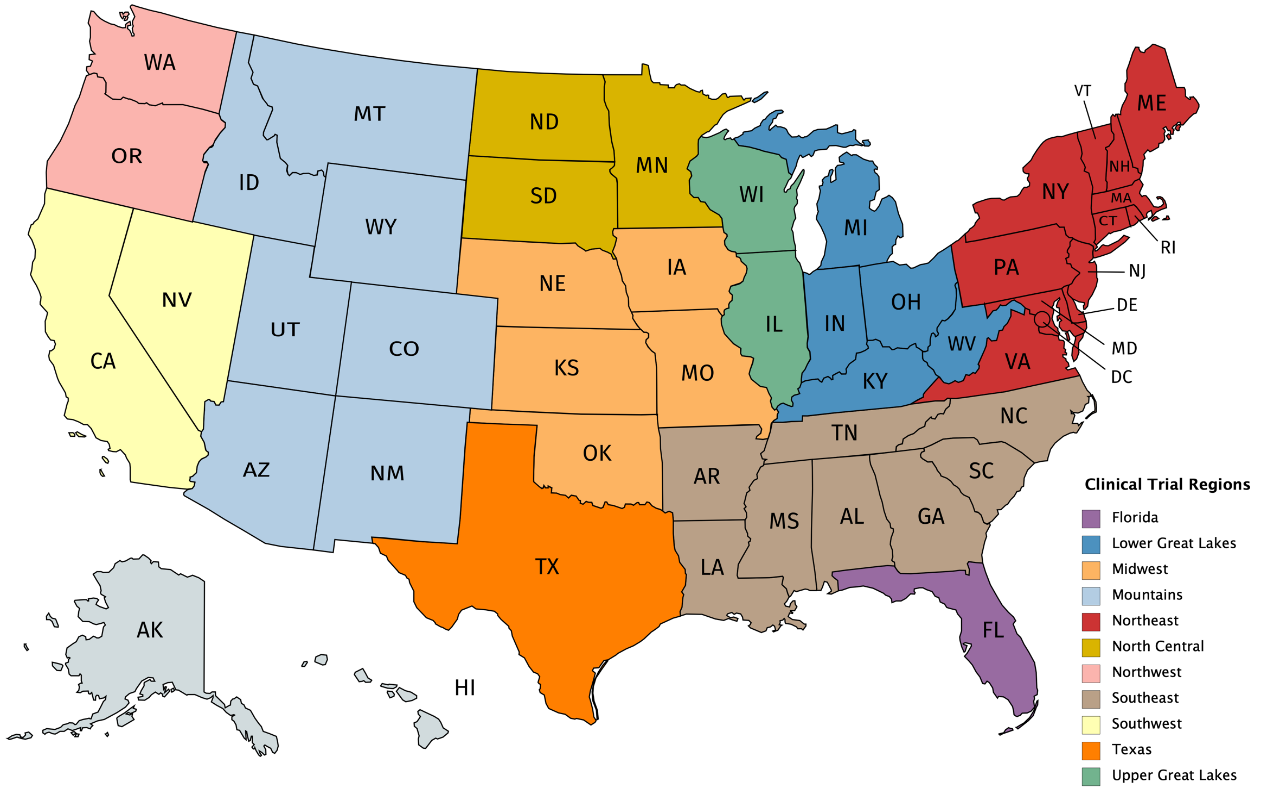

Created with mapchart.net

Supplemental Table 2. Research Clinic Types (N = 73)

Clinic Type	n	%
Academic medical center	14	19.2%
Community hospital	2	2.7%
Contract research organization	31	42.5%
Governmental organization	2	2.7%
Independent research clinic	22	30.1%
Pharmaceutical company	2	2.7%

Supplemental Table 3. Number of Healthy Volunteer Trials by Therapeutic Area (N = 1001)

Aggregate Therapeutic Area	n	%
Does not recall*	161	16.1%
Pain	98	9.8%
Cancer	67	6.7%
Autoimmune Diseases	53	5.3%
Hepatitis C	47	4.7%
Diabetes	45	4.5%
Psych-related	41	4.1%
Cholesterol	39	3.9%
Alzheimer’s disease	37	3.7%
Antibiotic	30	3.0%
Arthritis	26	2.6%
Brain-related	26	2.6%
Kidney-related	23	2.3%
Blood-related	22	2.2%
Multiple Sclerosis	22	2.2%
Lung-related	21	2.1%
Infectious disease	21	2.1%
Cancer-related	18	1.8%
HIV	17	1.7%
Liver-related	16	1.6%
Hypertension	16	1.6%
Parkinson’s disease	15	1.5%
Sleep-related	14	1.4%
Muscle-related	14	1.4%
Hormone-related	13	1.3%
Stomach-related	12	1.2%

Skin-related	10	1.0%
Allergies	9	0.9%
Heart-related	9	0.9%
Anti-Fungal	8	0.8%
Intestinal issues	8	0.8%
Fat-related	8	0.8%
Addiction	8	0.8%
Sexual-related	6	0.6%
Immunosuppressant	5	0.5%
STI	3	0.3%
Eye-related	3	0.3%
Bone-related	3	0.3%
Anesthetic	2	0.2%
Colonoscopy Screening Pill	2	0.2%
Hearing-related	1	0.1%
Pancreas-related	1	0.1%
Missing data	1	0.1%

* Of the studies in which the participant did not recall the therapeutic area of the clinical trial, 118 cases (73.3%) were studies for which the participant did not qualify for the trial or did not participate for some other reason.

Supplemental Table 4. Types of Included Study Procedures Reported by Participants in CTDs (N = 1001)

Procedures	n	% of trials
Blood draws	727	72.6%
ECGs	596	59.5%
Urine collection	424	42.4%
Holter monitor	90	9.0%
Genetic testing	70	7.0%
Stool collection	34	3.4%
Imaging/ CT scan / ultrasound / other radiology	27	2.7%
Lumbar puncture	9	0.9%
PET scan	8	0.8%
Endoscopy	5	0.5%
Semen collection	3	0.3%
Other*	49	4.9%

* Other was a write-in response and included procedures such as bronchoscopy, spirometry, electroencephalogram (EEG), and biopsy.
